# Laparoscopic Right Hemicolectomy With D2 Lymphadenectomy for Adult Ileocecal Intussusception Secondary to Cecal Adenocarcinoma: A Case Report

**DOI:** 10.7759/cureus.97702

**Published:** 2025-11-24

**Authors:** Alan Eduardo Soto Castro, Oscar Jesús Escoto Alemán, Teresa Rubi Soto Castro, Karen Celia Valadez Almaraz, Diego Delgado Gaheta, David Muñoz Ornelas, Jesús Ariel Andrade Lara, Josep May Rodriguez

**Affiliations:** 1 Surgery, Hospital Regional Instituto de Seguridad y Servicios Sociales de los Trabajadores del Estado (ISSSTE) Veracruz, Veracruz, MEX; 2 Surgery, Hospital General Regional No. 1 Instituto Mexicano del Seguro Social (IMSS), Ciudad Obregón, MEX; 3 Surgery, Universidad Latina de México, Celaya, MEX; 4 Faculty of Medicine, Universidad Autónoma de Nuevo León, Monterrey, MEX; 5 Surgery, Universidad Cristóbal Colón, Veracruz, MEX

**Keywords:** adult intussusception, d2 lymphadenectomy, ileocecal tumor, laparoscopic right hemicolectomy, minimally invasive surgery

## Abstract

Adult ileocecal intussusception is an uncommon condition that often indicates an underlying malignancy. A 47-year-old woman presented with a three-month history of bowel habit changes, hematochezia, abdominal pain, and a palpable epigastric mass. Laboratory evaluation showed anemia (hemoglobin 8.9 g/dL), thrombocytosis (529,000/μL), and elevated carcinoembryonic antigen (5.78 ng/mL). Contrast-enhanced CT revealed ileocecal intussusception extending into the transverse colon with pericolic lymphadenopathy. Diagnostic laparoscopy identified a 4 × 4 cm cecal mass and approximately 10 cm of intussuscepted ileum. Given the high suspicion of malignancy, no reduction was attempted. A laparoscopic right hemicolectomy with D2-level lymphadenectomy was performed, including pericolic and intermediate lymph node dissection along the ileocolic, right colic, and right branch of the middle colic vessels with ligation at the superior mesenteric artery origin. An end ileostomy was created due to anemia, transient vasopressor use, and mild bowel edema. The postoperative course was uneventful, with early ambulation, oral tolerance, and discharge after 48 hours. This case highlights the importance of maintaining oncologic principles in adult intussusception and demonstrates that laparoscopic right hemicolectomy with D2 lymphadenectomy is a safe, effective, and adaptable approach, even when temporary diversion is required.

## Introduction

Adult ileocecal (or ileocolic) intussusception is an uncommon clinical entity, representing only 1-5% of all bowel obstructions and approximately 5% of all intussusception cases [1]. In contrast to the idiopathic form typically seen in children, nearly 90% of adult cases have an identifiable pathological lead point, most often a neoplastic lesion [2]. Because of this high malignancy rate, current management principles emphasize en bloc oncologic resection without attempted reduction to avoid tumor dissemination and ensure adequate margins and lymph-node clearance [3].

Although the classification of lymphadenectomy levels (D1-D3) originates from Japanese gastric and colorectal oncologic surgery, it provides a useful framework for describing the extent of nodal dissection in colonic resections; In this setting, a D2 lymphadenectomy - encompassing pericolic and intermediate nodal basins with vascular ligation at the ileocolic origin - can achieve oncologic adequacy while minimizing operative trauma, particularly in emergency presentations where complete preoperative staging is not feasible [4]. 

Given the rarity of adult ileocecal intussusception and the limited literature describing standardized oncologic approaches - particularly the application of D2 lymphadenectomy and minimally invasive techniques in acute presentations - documenting this case provides valuable clinical insight. This report aims to highlight decision-making principles, operative considerations, and postoperative outcomes that may guide surgeons managing similar high-risk emergent scenarios.

## Case presentation

**Figure 1:**
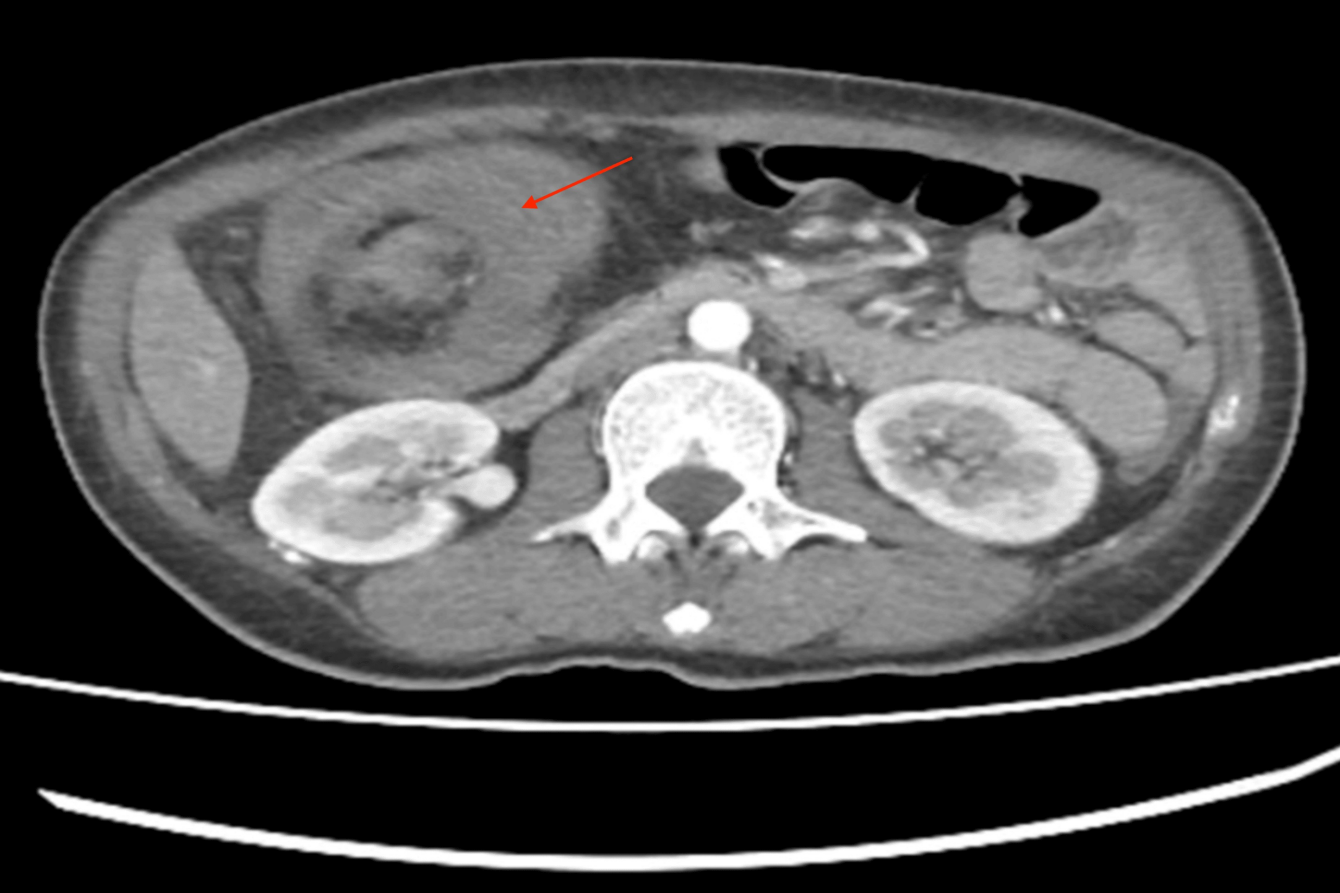
Pseudokidney sign: Bilobed image with an area of greater attenuation secondary to thickening of the intestinal wall identified at its periphery.

**Figure 2:**
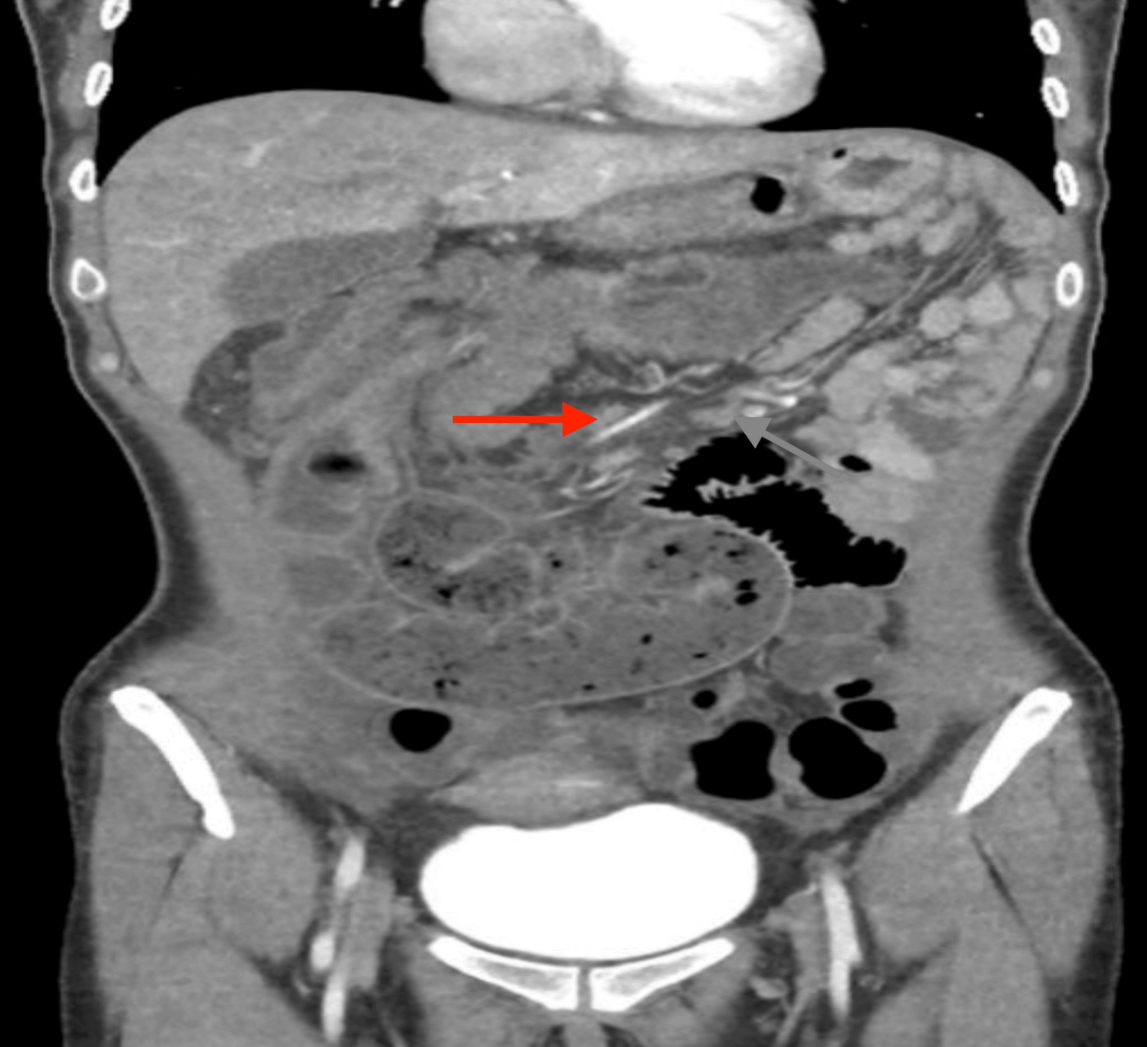
Transition zone: An abrupt decrease in the intestinal lumen with retrograde dilation of the small intestine loops is observed.

**Figure 3:**
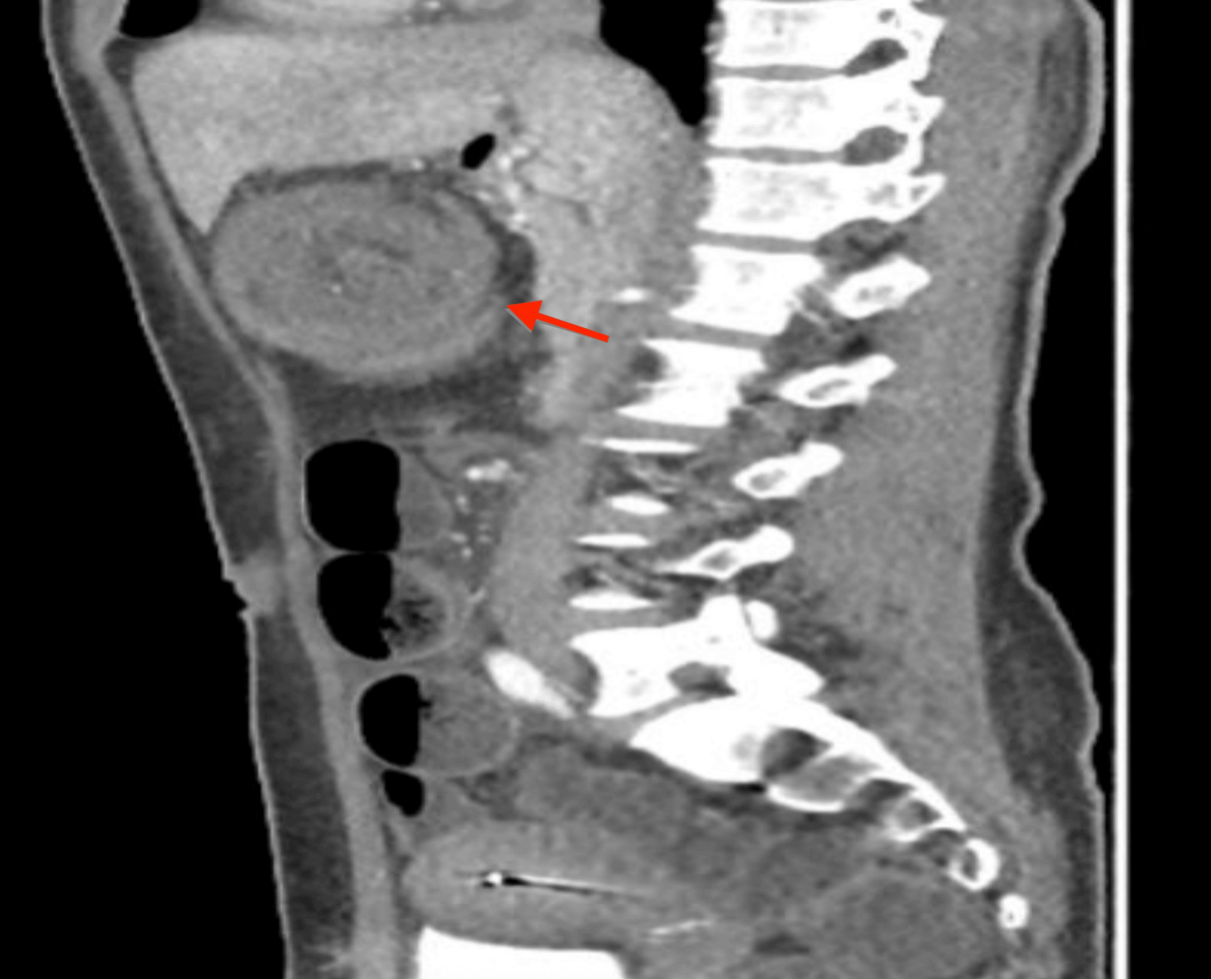
Crescent doughnut sign: Intraluminal identification of a lesion with soft-tissue density and eccentric fatty densities secondary to the invaginated mesentery.

**Figure 4:**
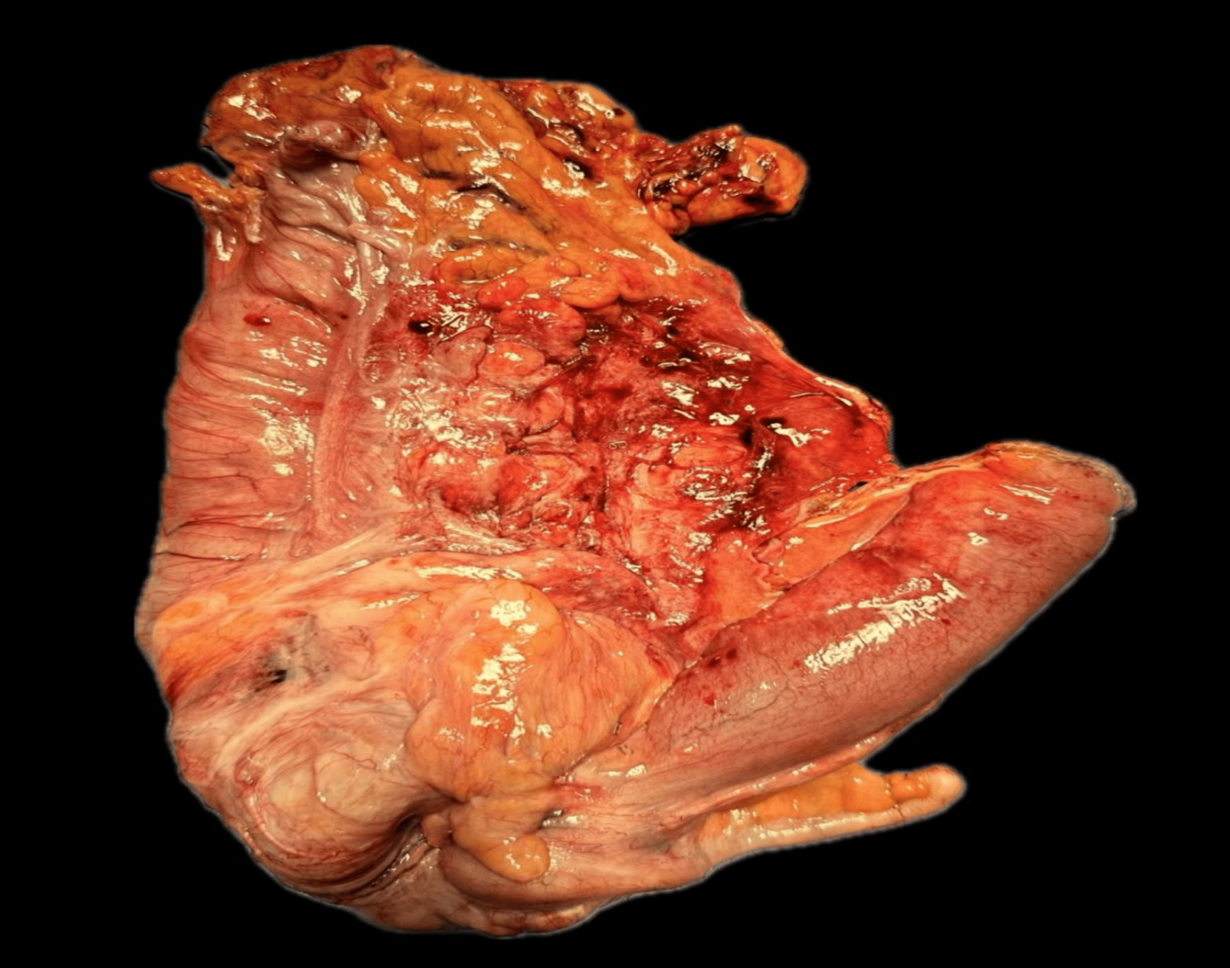
Cecal tumor: Measuring 4 × 4 centimeters, with multiple mesenteric lymphadenopathies up to 1 cm; the liver appears macroscopically without signs of tumor activity.

In March 2024, a 47-year-old female from Mexico City presented with a three-month history of bowel habit changes, hematochezia, intermittent colicky abdominal pain, a palpable epigastric mass, hyporexia, asthenia, adynamia, and a weight loss of 12 kg. Physical examination revealed a soft, compressible abdomen with a movable tumor in the epigastrium. Laboratory tests showed anemia (Hb 8.9 g/dL), thrombocytosis (529,000/μL), and a carcinoembryonic antigen level of 5.78 ng/mL. Contrast-enhanced abdominal CT (Figures 1-3) demonstrated ileocecal intussusception extending to the transverse colon with pericolic lymphadenopathies. Diagnostic laparoscopy revealed approximately 10 cm of ileocecal intussusception, a 4×4 cm cecal tumor, and multiple mesenteric lymph nodes up to 1 cm, without liver metastases (Figure 4).

Given intraoperative hemodynamic instability (mean arterial pressure (MAP) <65 mmHg requiring norepinephrine at 0.08 μg/kg/min for 25 minutes), chronic anemia (Hb 8.9 g/dL), hypoalbuminemia (2.9 g/dL), and bowel wall edema, primary anastomosis was considered high risk. Therefore, a laparoscopic right hemicolectomy with D2 lymphadenectomy and end ileostomy with stapled distal mucous fistula was performed.

Postoperatively, the patient resumed ambulation on day one, tolerated oral fluids within 12 hours, and achieved first flatus on day two. Pain was controlled with non-opioid analgesics. Ileostomy output averaged 800-1000 mL/day. No complications occurred (Clavien-Dindo 0). The patient met Enhanced Recovery After Surgery (ERAS) discharge criteria and was discharged after 48 hours, with no readmission at 30-day follow-up.

Final histopathology showed a moderately differentiated adenocarcinoma of the cecum (pT2N0, R0), with 18 lymph nodes examined - all negative for malignancy - and no evidence of lymphovascular or perineural invasion. Ileostomy reversal is planned at approximately three months, pending nutritional recovery and completion of oncologic assessment.

At follow-up, the patient remained clinically stable with no postoperative complications. At the two-week and four-week outpatient visits, the ileostomy demonstrated consistent function with adequate hydration and electrolyte balance. Nutritional status progressively improved, with weight stabilization and hemoglobin rising to 11.2 g/dL after supplementation. The patient reported good energy levels, normal oral intake, and no abdominal pain. The stoma reversal is planned for June 2024 following completion of nutritional optimization and routine oncologic evaluation. At the most recent follow-up (three months postoperatively), the patient remains asymptomatic and disease-free based on clinical assessment.

Intraoperatively, the decision to perform a terminal end ileostomy rather than primary anastomosis was based on the presence of multiple risk factors for anastomotic leak, including chronic anemia (hemoglobin 8.9 g/dL), transient vasopressor requirement to maintain mean arterial pressure above 65 mmHg, and mild bowel edema. Although intracorporeal anastomosis is generally favored for enhanced recovery, a conservative approach was chosen to optimize patient safety. Stoma reversal was planned following nutritional optimization and stabilization, approximately eight to 12 weeks postoperatively.

The postoperative recovery was uneventful (Clavien-Dindo grade 0). The patient achieved early ambulation within 12 hours, tolerated liquids on postoperative day one, and passed flatus within 24 hours. The ileostomy output averaged 600-800 mL per day, with no electrolyte imbalance or wound complications. The patient was discharged on postoperative day two under the institutional ERAS protocol, with follow-up scheduled for 30 days and oncology evaluation.

## Discussion

Ileocecal (or ileocolic) intussusception in adults is rare, representing only 1-5% of all intussusceptions and approximately 0.003-0.02% of hospital admissions [1]. Unlike pediatric cases, up to 90% of adult intussusceptions have an identifiable pathological lead point, most frequently a malignant neoplasm. Consequently, reduction is generally avoided when malignancy is suspected, and en bloc oncologic colectomy is recommended to prevent intraluminal dissemination or tumor seeding [2,3]. The operative strategy depends on anatomical site and etiology: small-bowel (enteroenteric) cases may allow limited resection if benign, whereas ileocolic or colocolic forms warrant right or left hemicolectomy, respectively, with lymphadenectomy according to oncologic principles [2,4].

In this case, no attempt at reduction was made intraoperatively due to the high index of suspicion for malignancy based on preoperative imaging and intraoperative findings. A laparoscopic right hemicolectomy with D2 lymphadenectomy was performed. In this context, D2 lymphadenectomy refers to the dissection and ligation of the ileocolic vessels at their origin from the superior mesenteric artery, including pericolic and intermediate lymph nodes along the ileocolic, right colic, and right branch of the middle colic vessels [4-6]. This corresponds to a standard oncologic right hemicolectomy or complete mesocolic excision (CME) with central vascular ligation (CVL) as described in Western surgical literature.

The feasibility of laparoscopic right hemicolectomy with oncologic lymphadenectomy has been demonstrated in several series, showing adequate lymph node yields (median >12 nodes), low conversion rates, and acceptable short-term morbidity [4-7]. The approach used - medial-to-lateral dissection with high vascular ligation - permits complete mesocolic mobilization while minimizing blood loss and preserving autonomic nerves.

The decision to avoid primary anastomosis is supported by existing evidence that identifies anemia, vasopressor use, and bowel edema as established risk factors for anastomotic leak [4-6]. Several series analyzing emergency right hemicolectomies have shown that diversion is an appropriate strategy in physiologically unstable patients or when tissue perfusion is uncertain, aligning with the decision-making in this case [4-6].

Furthermore, our patient’s rapid postoperative recovery is consistent with published outcomes of laparoscopic oncologic hemicolectomy demonstrating reduced postoperative pain, early mobilization, and shorter length of stay compared with open surgery [6-7]. Prior studies of D2 or CME/CVL resections have similarly reported low complication rates, validating the use of minimally invasive oncologic resection even in urgent settings [6-7].

This case underscores the importance of maintaining a high suspicion for malignancy in adult ileocecal intussusception and highlights the technical feasibility and safety of laparoscopic right hemicolectomy with D2-level lymphadenectomy as an effective oncologic approach in this clinical context [1-7].

## Conclusions

This case emphasizes that adult ileocecal intussusception should prompt strong suspicion for malignancy and warrants oncologic en bloc resection without attempted reduction. Laparoscopic right hemicolectomy with D2 lymphadenectomy proved safe and oncologically adequate in this emergency setting. A protective ileostomy was justified based on intraoperative risk factors and contributed to an uncomplicated recovery. Early diagnosis, adherence to oncologic principles, and individualized reconstruction strategies are key take-home lessons.
